# Preparation of Biphenyl-Conjugated Bromotyrosine for Inhibition of PD-1/PD-L1 Immune Checkpoint Interactions

**DOI:** 10.3390/ijms21103639

**Published:** 2020-05-21

**Authors:** Eun-Hye Kim, Masuki Kawamoto, Roopa Dharmatti, Eiry Kobatake, Yoshihiro Ito, Hideyuki Miyatake

**Affiliations:** 1 Nano Medical Engineering Laboratory, RIKEN Cluster of Pioneering Research, 2-1 Hirosawa, Wako, Saitama 351-0198, Japan; eunhye.kim@riken.jp (E.-H.K.); roopa.dharmatti@riken.jp (R.D.); y-ito@riken.jp (Y.I.); 2 Department of Life Science and Technology, School of Life Science and Technology, Tokyo Institute of Technology, 4259 Nagatsuta-cho, Midori-ku, Yokohama 226-8503, Japan; kobatake.e.aa@m.titech.ac.jp; 3 Emergent Bioengineering Materials Research Team, RIKEN Center for Emergent Matter Science, 2-1 Hirosawa, Wako, Saitama 351-0198, Japan

**Keywords:** PD-1/PD-L1, immune checkpoint inhibitors, biphenyl-conjugated bromotyrosine, amino acid conjugation, amino-X, in silico simulation, IC_50_

## Abstract

Cancer immunotherapy has been revolutionized by the development of monoclonal antibodies (mAbs) that inhibit interactions between immune checkpoint molecules, such as programmed cell-death 1 (PD-1), and its ligand PD-L1. However, mAb-based drugs have some drawbacks, including poor tumor penetration and high production costs, which could potentially be overcome by small molecule drugs. **BMS-8**, one of the potent small molecule drugs, induces homodimerization of PD-L1, thereby inhibiting its binding to PD-1. Our assay system revealed that **BMS-8** inhibited the PD-1/PD-L1 interaction with IC_50_ of 7.2 μM. To improve the IC_50_ value, we designed and synthesized a small molecule based on the molecular structure of **BMS-8** by in silico simulation. As a result, we successfully prepared a biphenyl-conjugated bromotyrosine (**X**) with IC_50_ of 1.5 μM, which was about five times improved from **BMS-8**. We further prepared amino acid conjugates of **X** (**amino-X)**, to elucidate a correlation between the docking modes of the **amino-X**s and IC_50_ values. The results suggested that the displacement of **amino-X**s from the **BMS-8** in the pocket of PD-L1 homodimer correlated with IC_50_ values. This observation provides us a further insight how to derivatize **X** for better inhibitory effect.

## 1. Introduction

Immunotherapy has recently emerged as a fourth modality for cancer therapy, together with surgery, chemotherapy, and radiation therapy [1,2,3,4]. The immunotherapy promotes T-cells to kill cancer cells by the blockade of immune checkpoint pathways [5,6]. One of the major immune checkpoint pathways is inactivated by the binding of programmed cell-death 1 (PD-1) [7], which is largely expressed on T cells, and its ligand PD-L1 [3,8,9], which is mainly expressed on antigen-presenting cells under physiological conditions but is upregulated on cancer cells [10]. PD-L1 binding to PD-1 suppresses T-cell function, including cytolytic activity, leading to downregulation of the anti-tumor immune response [2,5]. Another immune checkpoint is mediated by binding of the ligands B7-1/2 (CD80, CD86) on activated antigen-presenting cells or cancer cells to cytotoxic T-lymphocyte-associated protein 4 (CTLA-4) on T cells, which also suppresses T-cell activity [11,12]. Identification of these immunosuppressive pathways led to the development of monoclonal antibody (mAb)-based cancer therapies that inhibit PD-1/PD-L1 or CTLA-4/B7 pathways, thereby reinvigorating the host anti-tumor immune response [2,13,14,15,16,17]. Among the therapies currently approved for clinical use are the anti-CTLA-4 mAb ipilimumab (Yervoy^®^), which was the first immune checkpoint inhibitor to demonstrate an anti-cancer effect [18,19], and the anti-PD-1 mAb nivolumab (Opdivo^®^) [20]. In addition to these and other approved mAb-based immune checkpoint inhibitors [21], many others are currently in clinical trials for various cancers and immune-based diseases [22,23,24,25]. 

Protein-based drugs such as mAbs have some important drawbacks, such as high production costs associated with the preparation of biologicals [26], poor tumor penetration due to their large molecular weights (~150 kDa) [27], and unexpected post-translational glycosylation patterns [28]. Small molecule drugs, which are generally orally active and can overcome many of the challenges associated with protein drugs, are therefore being pursued as attractive alternative immune checkpoint inhibitors [28,29].

**Figure 1:**
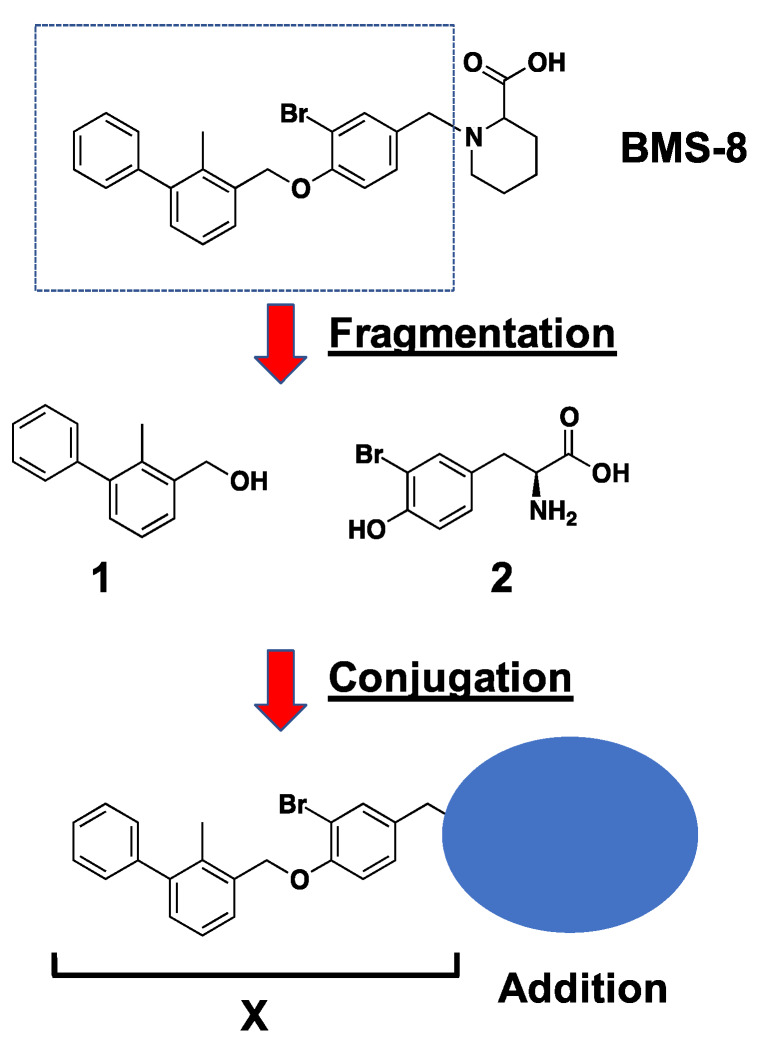
Strategies to improve inhibitory effect of **BMS-8**. 3-hydroxymethyl-2-methylbiphenyl (**1**) and 3-bromotyrosine (**2**) were selected as fragmented structures. A biphenyl-conjugated bromotyrosine **X** was synthesized after conjugation of **1** and **2**. We conjugated a variety of amino acids as additions, to the amino- and carboxyl-groups of **X** to reveal molecular mechanism of the inhibition.

Until now, Bristol-Myers Squibb (BMS) has disclosed the patent claim [30] with structures of a number of BMS compounds, which are the potential inhibitors of the PD-1/PD-L1 pathway. Previous works have shown that one of the BMS compounds, **BMS-8**, binds directly to PD-L1 and induces formation of PD-L1 homodimers, which in turn prevents the interaction with PD-1 [31]. In the patent claims, the homogenous time-resolved fluorescence (HTRF) assay report that **BMS-8** has a sub μM order of IC_50_, 0.146 μM [30], with other BMS compounds [32]. In this study, however, our amplified luminescence proximity homogeneous assay (Alpha) measured the IC_50_ of **BMS-8** as 7.2 μM. Therefore, we aimed to prepare higher affinity compounds by taking the advantage of the complex structure of **BMS-8**/PD-L1 [31] with in silico simulation [33,34,35]. Figure 1 shows our strategies to improve the affinity of **BMS-8**. We used fragmented structures of 3-hydroxymethyl-2-methylbiphenyl (**1**) and 3-bromotyrosine (**2**). After conjugation of **1** and **2**, a biphenyl-conjugated bromotyrosine (denoted as **X**) was synthesized. Because an amino and carboxyl group included in **X**, it could be conjugated to various amino acids. [36,37]. During the procedures, we employed in silico simulation and IC_50_ assay to reveal molecular mechanism of the inhibition.

## 2. Results

### 2.1. In Silico Docking Simulation and Organic Chemistry Synthesis of a Biphenyl-Conjugated Bromotyrosine

**Scheme 1:**
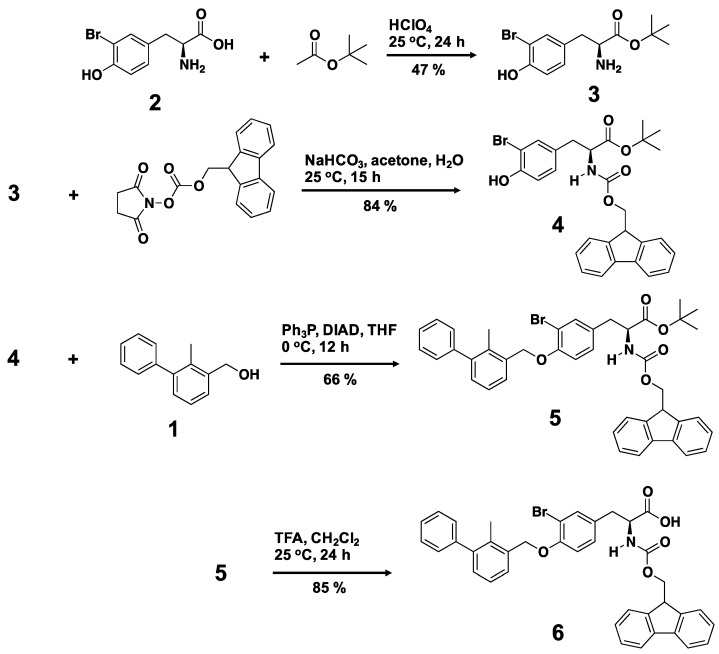
Synthetic scheme for the biphenyl-conjugated bromotyrosine **6**.

We designed a biphenyl-conjugated bromotyrosine (denoted as **X**), based on the **BMS-8**. We docked **X** into the crystal structure of **BMS-8**/PD-L1_AB_ complex (PDB ID: 5J8O) [31] using ICM 3.8-7 software (Molsoft L.L.C., San Diego, CA, USA) [33,34,35], without guidance and induced fitting to avoid over-fitting. We obtained the docking score of −42.96 for **X**, which was the same order of **BMS-8**, −49.5 (Table 1). Based on the scores, we confirmed the potential of **X** for inhibition. Therefore, we synthesized **X** by the organic chemistry procedures. Scheme 1 shows the synthetic route for a biphenyl-bromotyrosine **6**. Full synthesis details are provided in Materials and Methods. The C- and N-terminals of 3-bromotyrosine (**2**) were first protected by *tert*-butyl and fluorenylmethyloxycarbonyl (Fmoc) groups, respectively, to produce the amino acid **4**, which was then reacted with 3-hydroxymethyl-2-methylbiphenyl (**1**) through the Mitsunobu reaction to yield compound **5**. Deprotection of the *tert*-butyl group in compound **5** produced the Fmoc-protected amino acid **6**. Deprotection of the Fmoc group in **6** yielded the compound **X**. Peptide conjugates were obtained by solid-state peptide synthesis using compound **6**. ^1^H NMR spectra of the compounds are shown in Figures S1–S4. A summary of the analytical data for the synthesized compounds is given in Table S1. The analytical data indicate the successful synthesis of **X** and 29 **amino-X** derivatives consisting of 2-mers (**GX**, **XG**, **XS**, **XR**, **XA**, **XW**), 3-mers (**YXC**, **WXG**, **QXQ**, **CXA**, **RXN**, **SXR**, **NXR**, **CXR**, **GXG**, **XNL**, **XNH**, **XHP**, **XGG**), 4-mers (**XCSE**, **XGGG**), 5-mers (**WRXNN**, **ERXNK**, **WRXNQ**, **XRRRR**, **XGGGG**), 6-mer (**XGGGGG**), and 7-mers (**CERXNKM**, **FWRXNNI**).

### 2.2. Inhibition Assays of PD-1/PD-L1 Binding by BMS-8 and X

To evaluate the binding affinities of the compounds for PD-L1, we used the amplified luminescence proximity homogeneous assay (Alpha) by using the AlphaLISA^®^ assay kit [38]. This assay is based on photoinduced energy transfer between donor and acceptor beads conjugated to PD-1 and PD-L1, respectively (Figure S6).

**Figure 2:**
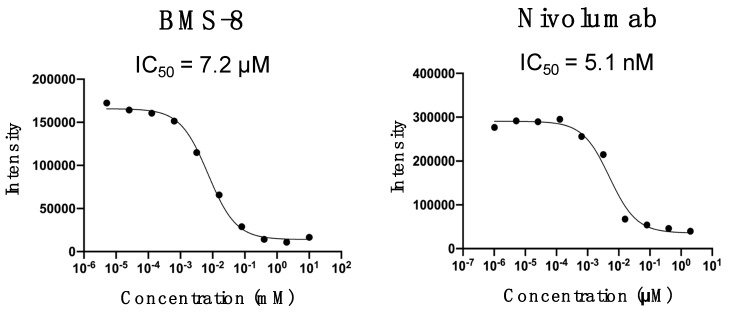
Inhibition of PD-1/PD-L1 interaction by **BMS-8** and nivolumab measured by the AlphaLISA^®^ assay.

**Figure 3:**
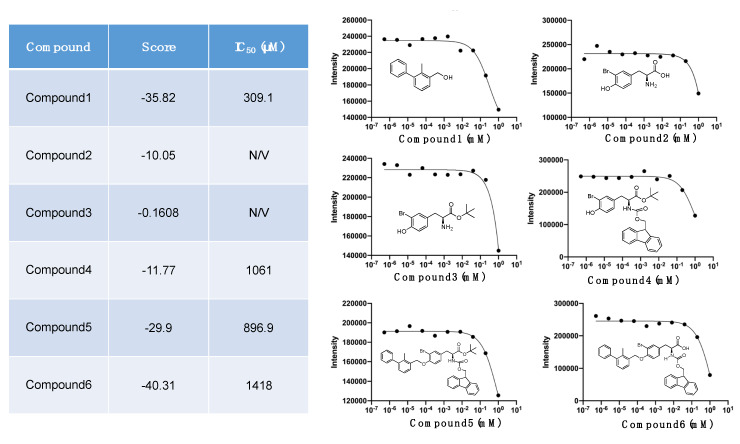
Docking scores, IC_50_ values and measurements of compounds **1–6**. All compounds showed larger scores than that of **X** (score = −42.96) with a few hundred μM of IC_50_ values.

The AlphaLISA^®^ assay revealed that the intermediates of **X**, compounds **1–6**, showed a few hundred μM or weaker IC_50_ values (Figure 3). **BMS-8** inhibited the PD-1/PD-L1 interaction with IC_50_ of 7.2 μM (Figure 2), which was weaker than that previously reported, IC_50_ of 0.146 μM [30]. On the other hand, nivolumab showed nano-molar order of inhibition (IC_50_ = 5.1 nM, Figure 2), corresponding to the previously reported value [39], which suggests the validity of our assay system. 

### 2.3. Fragmentation of BMS-8 and Conjugation of Compounds to Prepare X

To prepare higher affinity compounds based on **BMS-8**, we first considered a scenario that smaller groups of **BMS-8**, compounds **1**–**6** (Scheme 1), showed better inhibitory effect for PD-1/PD-L1 PPI. The docking scores of the compounds, however, were larger than that of **BMS-8** (−49.5), suggesting pooper inhibition effect. Actually, AlphaLISA assay revealed that the IC_50_ values were a few hundred μM, which were much weaker than that of **BMS-8** (7.2 μM) (Figure 3).

**Figure 4:**
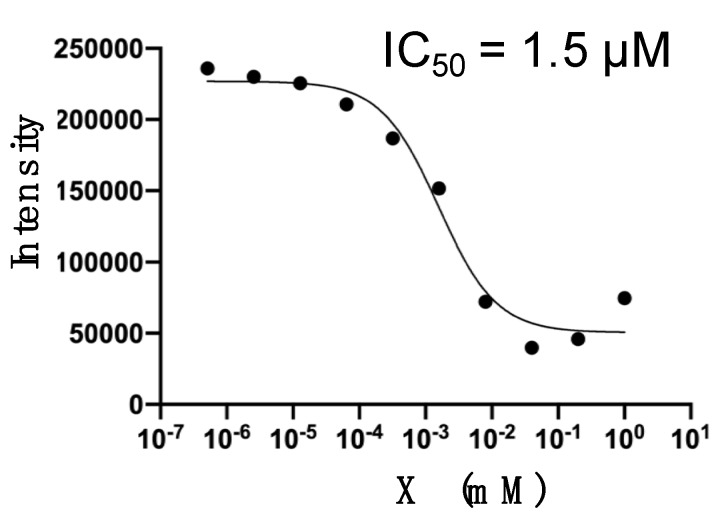
AlphaLISA assay of **X**. **X** shows IC_50_ = 1.5 μM with docking score = −42.96.

Therefore, we considered the next scenario of conjugation of compounds; we conjugated compound **4** and compound **1** to prepare biphenyl-bromotyrosine (**X**), which resembled **BMS-8** except the terminal amino- and carboxyl-groups. In turn, **X** showed a docking score of −42.96, comparable to that of **BMS-8** (−49.5). In fact, **X** inhibited PD-1/PD-L1 PPI with IC_50_ = 1.5 μM (Figure 4), which was five times better than that of **BMS-8** (7.2 μM).

### 2.4. Docking Simulation and Inhibition Assay of Amino-Xs

**Figure 5:**
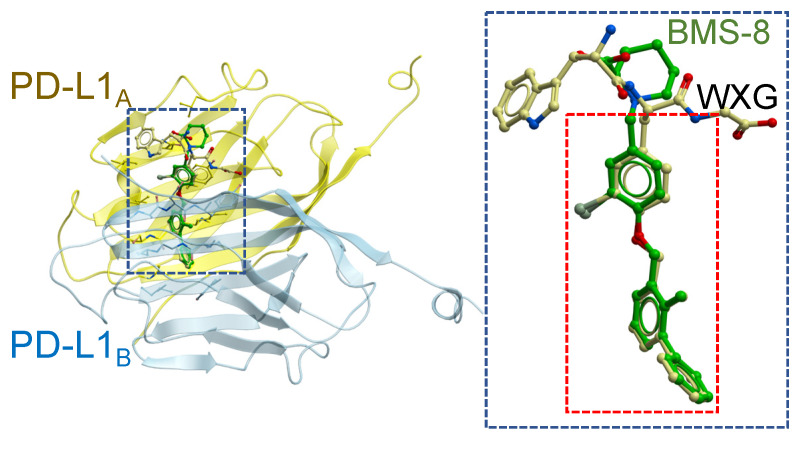
Root mean square deviation (RMSD) calculation between amino-X and **BMS-8** bound to PD-L1_AB_ homodimer. After docking of amino-X (in this case, WXG), we calculated RMSD between a part of **WXG** (excluding Cα, amino-group and carboxyl-group) and the corresponding part of **BMS-8**, as shown by the red dotted-rectangle.

The binding mode of the BMS compounds and derivatives to PD-L1 has previously been revealed by X-ray crystallography [31,40,41,42]. BMS compounds induces transient homodimerization of PD-L1_AB_ on the binding, which masks the binding site for PD-1 located in the homodimerization interface. We docked **amino-X**s to the crystal structure of **BMS-8**/PD-L1_AB_ complex (PDB ID: 5J8O) [31], using ICM 3.8-7 software (Molsoft L.L.C., San Diego, CA, USA) [33,34,35], without guidance and induced fitting to avoid over-fitting. After the docking, we calculated the root mean square deviation (RMSD) of distances between atoms in compound **BMS-8** and **X**, excluding Cα, NH_2_, and COOH atoms (Figure 5).

Table 1 shows the docking scores and RMSD values for **amino-X**s docked to PD-L1_AB_. Also, the IC_50_ values for the **amino-X**s are listed in Table 1. As a result, they suggested some positive correlations. The IC_50_ values of the 1–2-mer **amino-X**s showed moderate correlations with both the RMSDs (CC 0.67, Table 2) and the scores (CC 0.40, Table 2). However, these correlations weakened as the number of conjugated amino acids increased (RMSD from 0.67 to 0 and CC 0.40 to −0.20, Table 2). These results suggest that the current in silico docking worked better for **amino-X**s conjugated with shorter amino acids.

To discuss the correlations further, we compared the docking structures of **X** (IC_50_ = 1.5 μM), **XG** (IC_50_ = 2.1 μM), and **GX** (IC_50_ = 448.5 μM).

**Figure 6:**
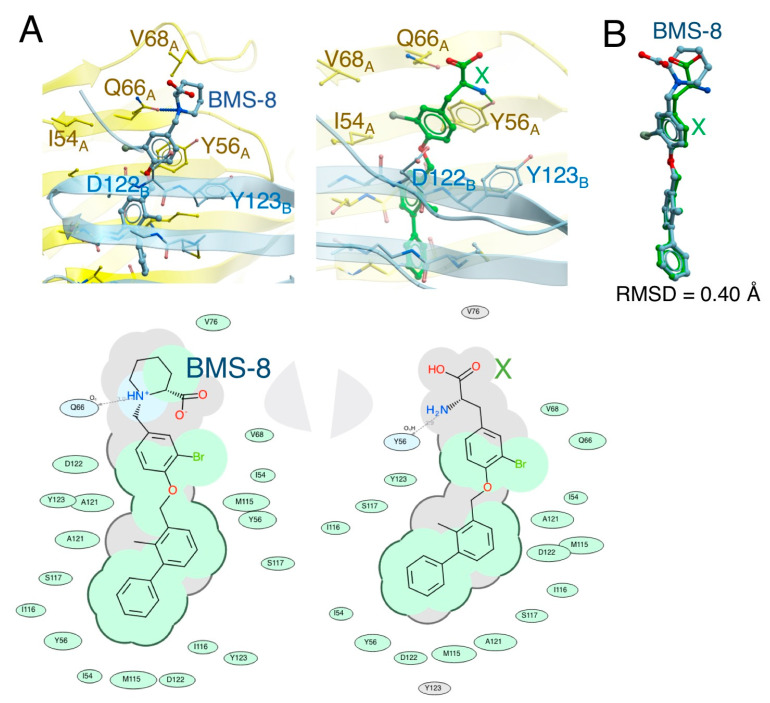
Docking conformations of **BMS-8** and **X**. (**A**) The docking modes of **BMS-8** and **X** were revealed by the X-ray crystallography and in silico docking simulation, respectively, which the 2D binding pictures. The 2D figures show that biphenyl portions of the ligands bind into the pocket by hydrophobic interactions shown in light-green color. In contrast, the amino cation at the six-membered ring of **BMS-8** makes a hydrogen bond with the sidechain of Q66_A_ in cyan color. In addition, the six-membered ring makes hydrophobic interaction with V68_A_. On the other hand, amino-group of bromo-tyrosine in **X** makes a hydrogen bonding to the hydroxyl group of Y56_A_ colored in cyan, without other hydrophobic interaction, as shown in the 2D picture. (**B**) **BMS-8** and **X** without Cα, NH_2_, and COOH superposed each other with RMSD of 0.40 Å.

We compared the binding modes of **BMS-8** and **X** in the pocket of PD-L1_AB_ homodimer (Figure 6). **BMS-8**, with IC_50_ of 7.2 μM (Figure 2), binds the pocket with a hydrogen bind to Q66_A_ and a hydrophobic interaction with V68_A_ (Figure 6A), respectively. On the other hand, **X** forms a hydrogen bond with the hydroxy group of the side chain of Y56_A_, which stabilizes the binding (Figure 6A), with IC_50_ of 1.5 μM (Figure 4). The superposition of **X** onto **BMS-8** showed an RMSD displacement of 0.40 Å (Figure 6B) We conclude that binding of **X** would not markedly impede PD-L1 homodimerization, which is consistent with its relatively low IC_50_ value of 1.5 μM (Figure 4). These results suggest that we can improve an IC_50_ value by substituting the six-membered group of **BMS-8** with some proper groups, leading to rearrangement of interactions around it. Besides, smaller displacement of biphenyl-bromotyrosine portion shown by RMSD is preferable for higher affinity.

**Figure 7:**
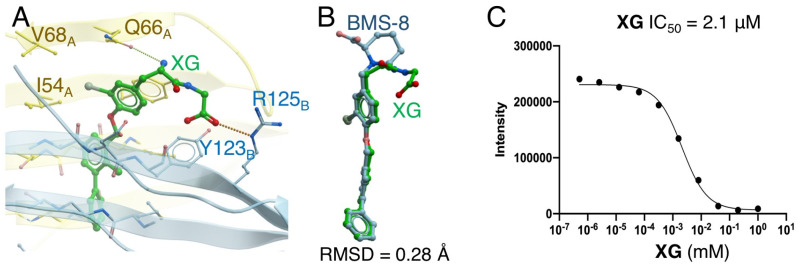
In silico binding mode of **XG**. (**A**) Behavior of **XG** in the binding pocket of the PD-L1_AB_ homodimer. (**B**) Superposition of **XG** onto **BMS-8**. The RMSD for displacement was 0.28 Å. (**C**) IC_50_ of **XG** for PD-1/PD-L1 binding.

Modeling of **XG** identified two potential hydrogen bonds between the N-terminal of **XG** and the side chain of Q66_A_ and between the carboxyl group of Gly and R125_B_ in the side chain (Figure 7A). The RMSD between **XG** and **BMS-8** was 0.28 Å (Figure 7B), which suggested that the IC_50_ value of **XG** would be similar to that of **X**. Indeed, **XG** had a measured IC_50_ for PD-1/PD-L1 binding of 2.1 μM (Figure 7C). **X** and **XG** potentially have the inhibitory effect for PD-1/PD-L1 interaction because K_D_ between PD-1 and PD-L1 are reported as 6.4 μM [43].

**Figure 8:**
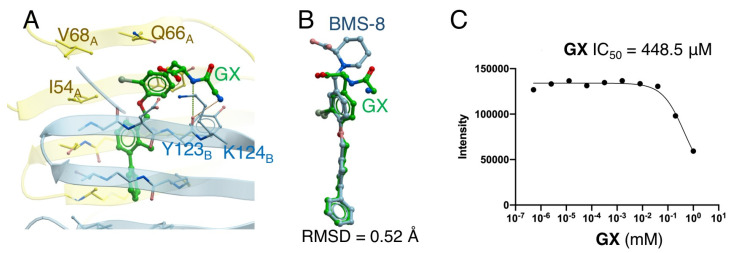
In silico binding mode of **GX**. (**A**) Behavior of **GX** in the binding pocket of the PD-L1_AB_ homodimer. (**B**) Superposition of **GX** onto **BMS-8**. The RMSD for displacement was 0.52 Å RMSD. (**C**) IC_50_ of **GX** for PD-1/PD-L1 binding.

**GX** docking into the binding pocket of the PD-L1 homodimer revealed two hydrogen bonds formed between **GX** amino groups and carbonyl group of Y123_B_ (Figure 8A). As a result, the calculated RMSD between **GX** and **BMS-8** was 0.52 Å (Figure 8B), which was larger than the RMSD of **X** and **XG**. This observation suggests that **GX** binding might sterically hinder PD-L1 homodimerization, leading to poorer inhibition of PD-1/PD-L1 binding. Consistent with this, the measured IC_50_ for **GX** was 448.5 μM (Figure 8C), which was several hundred times higher than those for **X** and **XG** ( Figure 4; Figure 7C). It is possible that the larger displacement of **X** of **GX** caused to deform the pocket of the PD-L1 homodimer, leading to the weaker inhibition of **GX** than those of **X** and **XG**.

**Figure 9:**
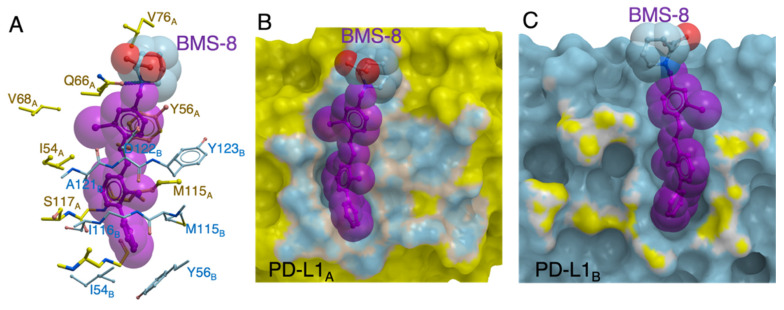
Schematic drawing and space-filling representation of **BMS-8** binding in the binding pocket of the PD-L1 homodimer. In (**A–C**), violet represents **X** without Cα, NH_2_, COOH atoms. (**A**) Binding mode of **BMS-8** in the pocket of the PD-L1_AB_ homodimer (PDB ID:5J8O). Yellow and cyan represent PD-L1_A_ and PD-L1_B_ side chains, respectively. (**B**) Space-filling representation of **BMS-8** bound to the surface of PD-L1_A_ (yellow) and contact area with PD-L1_B_ (cyan). (**C**) Space-filling representation of **BMS-8** bound to the surface of PD-L1_B_ (cyan) and contact area with PD-L1_A_ (yellow).

The **X** portion of **BMS-8** without Cα, NH_2_, COOH atoms formed hydrophobic interactions in the crystal structure (PDB ID: 5J8O), with residues I54_A_, Y56_A_, V68_A_, M115_A_, I116_A_, S117_A_, A121_A_, D122_A_, I54_B_, Y56_B_, M115_B_, I116_B_, S117_B_, A121_B_, D122_B_, and Y123_B_ of the PD-L1 homodimer (Figure 9A). The space-filling representation of **X** shows the adherent interaction mode to the binding pocket (Figure 9B,C). The intermediate compounds of **BMS-8**, compounds **1**–**6** (Scheme 1) showed a poor ability to inhibit PD-1/PD-L1 binding (Figure 3), which was probably due to insufficient hydrophobic filling of the compounds in the binding pocket.

Collectively, our results suggest that the larger displacement of **amino-Xs** from **BMS-8** prevents PD-L1_A_/PD-L1_B_ homodimer formation. The docking simulations suggest that **X** and **GX** promote homodimerization of PD-L1, resulting in low IC_50_ values, whereas the larger displacement of **amino-Xs** prevents PD-L1 homodimer formation and increase the IC_50_ values.

The results of this study advance our understanding of how small molecule compounds could be rationally designed to inhibit PD-1/PD-L1 interactions with high affinity. In silico docking simulations have typically shown that target proteins have stable binding pockets during ligand binding, even allowing for some local flexibility of the side chains within the pockets [37,44]. In that scenario, binding scores generally correlate well with experimentally determined inhibitor activity [45]. However, binding of **X** and **amino-X** in the PD-L1 pocket occurs through strict interactions, indicating that even a slight displacement of the **X** conformation leads to deformation of the PD-L1 homodimer, which deceases the inhibitory effect. Consistent with this, the **amino-X**s with shorter amino acid conjugates showed moderate positive correlations between the measured IC_50_ values and RMSDs in the no template/flexible docking mode, whereas the correlation was weakened by further amino acid addition.

## 3. Materials and Methods

### 3.1. Materials for Organic Chemistry Synthesis

Sodium chloride (NaCl), lysozyme, monosodium phosphate (NaH_2_PO_4_), imidazole, glycerol, reduced glutathione, oxidized glutathione, methanol, dimethyl sulfoxide (DMSO), trifluoroacetic acid (TFA), tert-butyl acetate, perchloric acid (HClO_4_), hydrochloric acid (HCl), sodium carbonate, ethyl acetate, sodium sulfate, hexane, sodium hydrogen carbonate (NaHCO_3_), acetone, triphenyl phosphine (Ph_3_P), anhydrous dichloromethane (CH_2_Cl_2_), and anhydrous tetrahydrofuran (THF) were purchased from Wako Pure Chemical Industries Ltd. (Osaka, Japan). 3-Bromo-tyrosine, 3-hydroxymethyl-2-methylbiphenyl, and diisopropyl azodicarboxylate (DIAD; 40% in toluene, approximately 1.9 mol L^−1^) were purchased from Tokyo Chemical Industry Co., Ltd. (Tokyo, Japan). Magnesium sulfate and CH_2_Cl_2_ were purchased from Junsei Chemical Co., Ltd. (Tokyo, Japan). Deuterochloroform (CDCl_3_) was purchased from Isotec, Inc. (Miamisburg, OH, USA), and *N*-[(9H-fluoren-9-ylmethoxy) carbonyloxy] succinimide (Fmoc-Osu) was purchased from Watanabe Chemical Industries, Ltd. (Hiroshima, Japan).

### 3.2. Synthesis of a Biphenyl-Conjugated Bromotyrosine


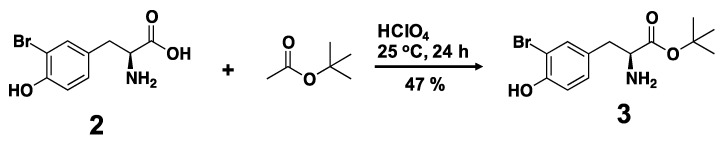


**(S)-tert-Butyl 2-amino-3-(3-bromo-4-hydroxyphenyl) propanoate (3)**. A suspension of 3-bromotyrosine (**2**; 1.0 g, 3.9 mmol) in tert-butyl acetate (16 mL, 92 mmol) was cooled to 0 °C, and stirred for 30 min. HClO_4_ (0.5 mL, 7.7 mmol) was then slowly added to the suspension at 0 °C, and the reaction mixture was warmed to 25 °C and stirred for 24 h. The mixture was washed with water and 1N HCl, and the aqueous phase was brought to pH 9 using sodium carbonate and then extracted with ethyl acetate. The resulting organic phase was washed with water and dried with sodium sulfate. The solvent was evaporated under reduced pressure, yielding an oily compound. This crude product was washed with cold hexane and then dried under reduced pressure to yield compound **3** (0.57 g, 47%). ^1^H-NMR (400 MHz, CDCl_3_): δ = 1.41 (s, 9H, –OC(CH_3_)_3_), 2.73 (dd, 1H, J = 14.4, 8.0 Hz, HOPh(Br)–CH_2_CH(NH_2_)–), 2.93 (dd, 1H, J = 13.6, 5.2 Hz, HOPh(Br)–CH_2_CH(NH_2_)–), 3.57 (dd, 1H, J = 7.2, 5.6 Hz, HOPh(Br)–CH_2_CH(NH_2_)–), 3.70 (m, 3H, HOPh(Br)–CH_2_CH(NH_2_)–), 6.70 (d, 1H, J = 8.0 Hz, aromatic ring), 6.94 (dd, 1H, J = 8.4, 2.0 Hz, aromatic ring), 7.26 (d, 1H, J = 1.6 Hz, aromatic ring).


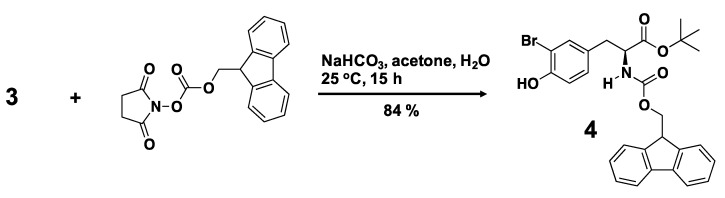


**(S)-tert-Butyl 2-({[(9H-fluoren-9-yl)methoxy]carbonyl}amino)-3-(3-bromo-4-hydroxyphenyl)propanoate (4)**. A suspension of **3** (0.5 g, 1.6 mmol) and NaHCO_3_ (0.27 g, 3.2 mmol) in water (20 mL) was cooled to 0 °C. Fmoc-Osu (1.1 g, 3.2 mmol) in acetone (40 mL) was added to the suspension slowly, and the reaction mixture was then stirred at 25 °C for 15 h. The solvent was removed and washed with 1N HCl and water. After drying under vacuum, the crude product was purified by column chromatography on silica gel (eluent: ethyl acetate/hexane = 1:3 v/v) to yield compound **4** (0.71 g, 84%). ^1^H-NMR (400 MHz, CDCl_3_): δ = 1.42 (s, 9H, –OC(CH_3_)_3_), 3.00 (d, 2H, J = 5.6 Hz, HOPh(Br)–CH_2_CH(NHCOOCH_2_CH–)–), 4.21 (t, 1H, J = 7.2 Hz, HOPh(Br)–CH_2_CH(NHCOOCH_2_CH–)–), 4.33 (dd, 1H, J = 10.4, 7.2 Hz, HOPh(Br)–CH_2_CH(NHCOOCH_2_CH–)–), 4.43–5.00 (m, 2H, HOPh(Br)–CH_2_CH(NHCOOCH_2_CH–)–), 5.29 (d, 1H, J = 8.0 Hz, HOPh(Br)–CH_2_CH(NHCOOCH_2_CH–)–), 5.43 (s, 1H, HOPh(Br)–CH_2_CH(NHCOOCH_2_CH–)–), 6.91 (d, 1H, J = 8.4 Hz, aromatic ring), 6.96 (d, 1H, J = 9.2 Hz, aromatic ring), 7.26–7.33 (m, 3H, aromatic ring), 7.40 (dd, 2H, J = 7.4, 7.4 Hz, aromatic ring), 7.57 (dd, 2H, J = 6.2, 6.2 Hz, aromatic ring), 7.76 (d, 2H, J = 7.2 Hz, aromatic ring); high resolution mass spectrometry (HRMS) calculated for C_28_H_28_BrNO_5_ ([M + H]^+^): 538.1224, found: 538.1224.


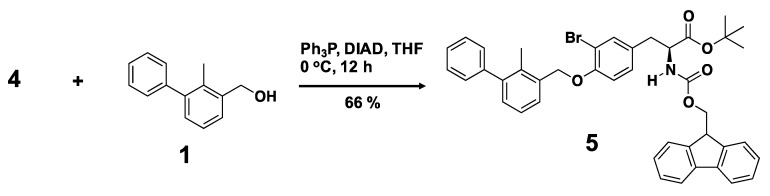


**(S)-tert-Butyl 2-({[(9H-fluoren-9-yl)methoxy]carbonyl}amino)-3-{3-bromo-4-[(2-methyl-1,1’-biphenyl-3-yl)methoxy]phenyl}propanoate (5).** To a solution of **4** (0.1 g, 0.19 mmol), 3-hydroxymethyl-2-methylbiphenyl (1; 39 mg, 0.20 mmol), and triphenyl phosphine (57 mg, 0.20 mmol) in anhydrous THF (10 mL), DIAD (0.1 mL, 0.22 mmol) was added at 0 °C under argon, and the reaction mixture was stirred at 0 °C for 12 h under argon. The organic phase was extracted with CH_2_Cl_2_ and dried over anhydrous magnesium sulfate. The solvent was then evaporated under reduced pressure, with the temperature kept below 30 °C. The crude product was purified by column chromatography on silica gel (eluent: ethyl acetate/hexane = 1:4 v/v) to yield compound **5** (0.09 g, 66%). ^1^H-NMR (400 MHz, CDCl_3_): δ = 1.46 (s, 9H, –OC(CH_3_)_3_), 2.27 (s, 3H, Biphenyl(CH_3_)–CH_2_OPh(Br)–CH_2_CH(NHCOOCH_2_CH–)–), 3.05 (d, 2H, J = 5.6 Hz, Biphenyl(CH_3_)–CH_2_OPh(Br)–CH_2_CH(NHCOOCH_2_CH–)–), 4.23 (t, 1H, J = 7.6 Hz, Biphenyl(CH_3_)–CH_2_OPh(Br)–CH_2_CH(NHCOOCH_2_CH–)–), 4.34 (dd, 1H, J = 6.8, 6.8 Hz, Biphenyl(CH_3_)–CH_2_OPh(Br)–CH_2_CH(NHCOOCH_2_CH–)–), 4.46-4.56 (m, 2H, Biphenyl(CH_3_)–CH_2_OPh(Br)–CH_2_CH(NHCOOCH_2_CH–)–), 5.13 (s, 2H, Biphenyl(CH_3_)-CH_2_OPh(Br)-CH_2_CH(NHCOOCH_2_CH–)–), 5.37 (d, 1H, J = 7.6 Hz, Biphenyl(CH_3_)–CH_2_OPh(Br)-CH_2_CH(NHCOOCH_2_CH–)–), 6.92 (d, 1H, J = 8.0 Hz, aromatic ring), 7.05 (d, 1H, J = 7.2 Hz, aromatic ring), 7.19–7.45 (m, 14H, aromatic ring), 7.53 (d, 1H, J = 6.8 Hz, aromatic ring), 7.60 (dd, 2H, J = 6.4, 6.4 Hz, aromatic ring), 7.77 (d, 2H, J = 7.2 Hz, aromatic ring); HRMS calculated for C_42_H_40_BrNO_5_ ([M + H]^+^): 718.2163, found: 718.2164.


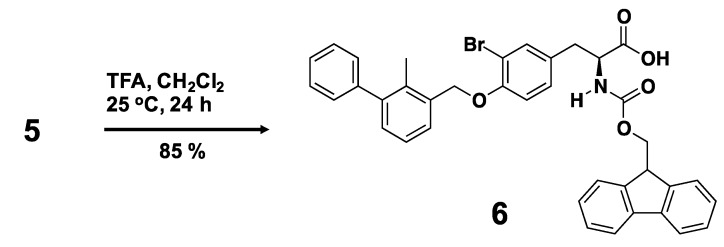


**(S)-2-({[(9H-fluoren-9-yl)methoxy]carbonyl}amino)-3-{3-bromo-4-[(2-methyl-1,1’-biphenyl-3-yl)methoxy]phenyl}propanoic acid (6)**. A solution of **5** (3.9 g, 5.42 mmol) in anhydrous CH_2_Cl_2_ (36 mL) was stirred at 0 °C under argon for 15 min. TFA (1.3 mL, 16.6 mmol) was added dropwise to the solution at 0 °C, and the reaction mixture was stirred at 25 °C under argon. After 6 h, TFA (1.5 mL, 19.5 mmol) was added to the reaction mixture, which was then stirred at 25 °C for 18 h under argon. The solvent was removed under reduced pressure, with the temperature kept below 40 °C. The crude product was purified by column chromatography on silica gel (eluent: CH_2_Cl_2_/methanol = 97:3 v/v) to yield compound **6** (3.2 g, 85%). ^1^H-NMR (400 MHz, CDCl_3_): δ = 2.24 (s, 3H, Biphenyl(CH_3_)–CH_2_OPh(Br)–CH_2_CH(NHCOOCH_2_CH–)–), 3.05 (dd, 1H, J = 14.0, 6.0 Hz, Biphenyl(CH_3_)–CH_2_OPh(Br)–CH_2_CH(NHCOOCH_2_CH–)–), 3.15 (dd, 1H, J = 14.8, 5.2 Hz, Biphenyl(CH_3_)–CH_2_OPh(Br)–CH_2_CH(NHCOOCH_2_CH–)–), 4.21 (t, 1H, J = 6.8 Hz, Biphenyl(CH_3_)–CH_2_OPh(Br)–CH_2_CH(NHCOOCH_2_CH–)–), 4.36 (dd, 1H, J = 6.8, 6.8 Hz, Biphenyl(CH_3_)–CH_2_OPh(Br)–CH_2_CH(NHCOOCH_2_CH–)–), 4.46 (dd, 1H, J = 10.0, 7.2 Hz, Biphenyl(CH_3_)–CH_2_OPh(Br)–CH_2_CH(NHCOOCH_2_CH–)–), 4.66 (dd, 1H, J = 13.2, 6.0 Hz, Biphenyl(CH_3_)–CH_2_OPh(Br)–CH_2_CH(NHCOOCH_2_CH–)–), 5.09 (s, 2H, Biphenyl(CH_3_)-CH_2_OPh(Br)–CH_2_CH(NHCOOCH_2_CH–)–), 5.23 (d, 1H, J = 8.4 Hz, Biphenyl(CH_3_)-CH_2_OPh(Br)–CH_2_CH(NHCOOCH_2_CH–)–), 6.91 (d, 1H, J = 8.8 Hz, aromatic ring), 7.03 (d, 1H, J = 7.6 Hz, aromatic ring), 7.21–7.55 (m, 15H, aromatic ring), 7.74 (d, 2H, J = 7.2 Hz, aromatic ring); HRMS calculated for C_38_H_32_BrNO_5_ ([M + H]^+^): 662.1537, found: 662.1520.

### 3.3. Solid-State Peptide Synthesis

**Amino-X**s were synthesized using an automated peptide synthesizer (MultiPep CF, INTAVIS Bioanalytical Instruments AG, Cologne, Germany). The synthetic protocol for glycine-conjugated peptide **XG** was as follows: Fmoc-protected glycine attached to a polystyrene resin (Fmoc-Gly NovaSyn TGT, Merck KGaA, Darmstadt, Germany) was deprotected by piperidine (20% in *N*-methylpyrrolidone (NMP). The resulting resin was reacted with **6** (99 mg, 0.14 mmol), 1-[bis(dimethylamino)methylene]-1H-benzotriazolium 3-oxide hexafluorophosphate (HBTU; 150 μL, 0.5 M in *N,N*-dimethylformamide (DMF), *N*-methylmorpholine (45 μL, 4.0 M in DMF) in NMP (8 μL) for 45 min. After washing, the *N*-α-protecting group of Fmoc in compound **6** was deprotected by piperidine (20% in NMP). Finally, the obtained peptide was cleaved from the resin using TFA (95% in water), yielding **XG**. Other peptides were synthesized using a similar method. (*S*)-2-amino-3-[3-bromo-4-{(2-methyl-1,1′-biphenyl-3-yl)methoxy}phenyl]propanoic acid (**X**) was obtained by deprotection of Fmoc in **6** using piperidine (20% in NMP).

### 3.4. Characterization

The synthesized compounds were identified using ^1^H NMR spectroscopy (JNM–ECZ400R, JEOL Ltd., Tokyo, Japan) and HRMS (QSTAR Elite, AB SCIEX, Framingham, MA, USA).

### 3.5. Determination of the IC_50_ Value by AlphaLISA^®^

#### 3.5.1. Principle of the Competitive Binding Assay

The binding affinity of the inhibitors to PD-L1 were measured using the AlphaLISA^®^ assay kit (AL356 HV/C/F, PerkinElmer) according to the manufacturer’s instructions, with the anti-PD-1 mAb nivolumab (Selleck Chemicals, Houston, TX, USA) included as a positive control [41]. In this assay, direct binding of an inhibitor to PD-L1 is detected by photoinduced energy transfer (Figure S6). Biotin-conjugated PD-1 is attached to streptavidin-coated donor beads and histidine (His)-tagged PD-L1 is attached to anti-His-conjugated acceptor beads. Photoexcitation of the donor beads at 680 nm yields singlet oxygen. If PD-L1–PD-1 binding is successful, energy is transferred through singlet oxygen, leading to an increase in fluorescence intensity at 615 nm (Figure S6).

#### 3.5.2. Preparation of Samples

**BMS-8** was purchased from AA Blocks LLC (San Diego, CA, USA). Stock solutions of inhibitors in DMSO (stock solution A, 5 mM) were serially diluted (Figure S5A) to obtain 10 assay solutions (1–10) with concentrations ranging from 5.0 mM to 2.6 nM (Table S2). An aliquot of solution 1–10 (2 μL) was mixed with His-tagged PD-L1 (25 nM, 2 μL), biotin-conjugated PD-1 (25 nM, 2 μL), anti-His acceptor beads (0.55 g L^–1^, 2 μL), and streptavidin-coated donor beads (1.1 g L^−1^, 2 μL) (Figure S5B) in a final volume of 10 μL and incubated at 25 °C for 90 min. Positive and negative technical controls were included in parallel. Positive controls contained buffer (2 μL) in place of solution 1–10, and negative controls contained only the beads (2 μL each) and buffer (6 μL).

#### 3.5.3. AlphaLISA^®^ Measurement and Analysis

The reaction samples (10 μM) were placed in a 384-well microplate and photoirradiated at 680 nm from the top. Fluorescence at 615 nm was detected using an EnSpire multimode plate reader (Perkin Elmer, Waltham, MA, USA). IC_50_ values were estimated from a sigmoidal curve of fluorescence intensity vs. inhibitor concentration using a relative weighting method (1/Y^2^ weighting) with GraphPad Prism 8 (GraphPad Software Inc., San Diego, CA, USA).

### 3.6. Docking Simulation of Compounds

The docking simulation software ICM 3.8-7 [33] was used to investigate the binding modes of **X** and **amino-X**s to the PD-L1 homodimer complexed with **BMS-8** (PDB ID: 5J8O) [31]. We performed docking without template docking [37] or introducing flexibility [37] to avoid over-fitting of the ligands into the pocket. The docking simulation supposed Monte Carlo pseudo-Brownian motion [46]. In the simulation, the score suggests goodness of docking, defined as follows [45]:

Score = Δ*E*_IntFF_ + *T*Δ*S*_Tor_ + α_1_Δ*E*_HBond_ + α_2_Δ*E*_HBDesol_ + α_3_Δ*E*_solEl_ + α_4_Δ*E*_HPob_ + α_5_*Q*_size_ (1)

where α_1_–α_5_ = weight, Δ*E*_IntFF_ = ligand–target van der Waals interactions and internal force field energy of the ligand, *T*Δ*S*_Tor_ = free energy changes due to conformational energy loss upon ligand binding, Δ*E*_HBond_ = hydrogen bonding interactions, Δ*E*_HBDesol_ = hydrogen bond donor–acceptor desolvation energy, Δ*E*_solEl_ = solvation electrostatic energy upon ligand binding, Δ*E*_HPob_ = hydrophobic free energy gain, and *Q*_size_ = a size correction term proportional to the number of ligand atoms [45,47,48]. We calculated RMSD values by using CORREL function in the Microsoft Excel.

## 4. Conclusions

This study reports that we prepared the new biphenyl-conjugated bromotyrosine, which inhibits the PD-1/PD-L1 interaction with better effect than that of **BMS-8**. In addition, the **amino-X**s, which are conjugates of **X** with a variety of amino acids, provide the molecular mechanism how amino acid modifications of **X** affects inhibition of PD-1/PD-L1 interactions. Binding of the **X** without the Cα, NH_2_, and COOH atoms portion of **amino-X**s into the PD-L1 binding pocket is required to promote transient homodimerization of PD-L1_A_/PD-L1_B_, leading to formation of a stable ternary complex composed of **X** and PD-L1_AB_. Amino acid conjugation, however, alters the **X** docking conformation in the PD-L1 pocket, reducing the IC_50_ values dramatically. We conclude that improper interactions between amino acids conjugated to **X** and those in the binding pocket induced displacement of the compounds, thereby reducing inhibitory effect. In the future, we plan to design conjugates with amino acids that do not disturb the conformation of **X** in the PD-L1 binding pocket.

## Figures and Tables

**Table 1:** Docking simulation and IC_50_ measurements of **BMS-8** and **amino-X**s.

Amino Acid Length	Sequence	Score	RMSD (Å)	IC_50_ (μM)
-	**BMS-8**	−49.5	-	7.2
**1**	**X**	−42.96	0.40	1.5
**2**	**GX**	−41.0	0.52	448.5
	**XG**	−46.9	0.28	2.1
	**XS**	−42.1	0.60	2655.0
	**XR**	−45.7	0.37	892.0
	**XA**	−43.1	0.47	22.3
	**XW**	−43.3	0.51	845.0
**3**	**YXC**	−37.1	0.46	465.0
	**WXG**	−50.6	0.48	404.8
	**QXQ**	−37.7	0.73	1961.0
	**CXA**	−42.0	0.48	665.0
	**RXN**	−40.3	0.63	405.3
	**SXR**	−36.7	0.58	796.0
	**NXR**	−50.3	0.46	982.0
	**CXR**	−41.5	0.54	550.0
	**GXG**	−43.6	0.39	676.0
	**XNL**	−43.0	0.58	855.0
	**XNH**	−40.7	0.50	313.0
	**XHP**	−33.5	0.55	359.0
	**XGG**	−36.1	0.57	6505.0
**4**	**XCSE**	−32.6	0.45	1555.0
	**XGGG**	−53.3	0.51	6766.0
**5**	**WRXNN**	−38.1	0.38	157.4
	**ERXNK**	−21.3	0.48	15.6
	**WRXNQ**	−19.4	0.49	163.2
	**XRRRR**	−28.3	0.45	435.6
	**XGGGG**	−41.8	0.75	647.5
**6**	**XGGGGG**	−45.3	0.48	846.0
**7**	**CERXNKM**	4.65	1.80	308.2
	**FWRXNNI**	−7.30	0.41	311.8

**Table 2:** Correlation coefficients (CC) for IC_50_ vs. Score and IC_50_ vs. RMSD.

Length	CC of Score/IC_50_	CC of RMSD/IC_50_
**1-2**	0.40	0.67
**1-3**	0.35	0.37
**1-4**	0	0.28
**1-7**	−0.20	0

CC values were calculated by the Microsoft Excel.

## Supplementary Materials

The following are available online at https://www.mdpi.com/1422-0067/21/10/3639/s1.

## Abbreviations

PD-1	Programmed cell death 1
PD-L1	Programmed cell death-ligand 1
PD-L1_A_	PD-L1 chain A
PD-L1_B_	PD-L1 chain B
PD-L1_AB_	Homodimer of PD-L1_A_/PD-L1_B_ chains
Alpha	Amplified Luminescence Proximity Homogeneous Assay
**X**	biphenyl-conjugated bromotyrosine
**Amino-X**	Amino acid conjugated-**X**
MALDI-TOF MS	Matrix assisted laser desorption/ionization-time of flight mass spectrometry
RMSD	Root mean square deviation
K_D_	Equilibrium dissociation constant
IC_50_	50% maximal inhibitory concentration
CC	Correlation coefficient
HTRF	Homogenous Time-Resolved Fluorescence
