# Supplementary material for: Preparation of Biphenyl-Conjugated Bromotyrosine for Inhibition of PD-1/PD-L1 Immune Checkpoint Interactions
Source: Int J Mol Sci. 2020 May 21;21(10):3639. doi: 10.3390/ijms21103639 (PMC7279355; doi:10.3390/ijms21103639)
Supplement: Supplementary file 1 [file ijms-21-03639-s001.pdf]

Preparation of Biphenyl-Conjugated Bromotyrosine for Inhibition of PD-1/PD-L1 Immune Checkpoint Interactions

Eun-Hye Kim [1,2], Masuki Kawamoto [1,3,*], Roopa Dharmatti [1], Eiry Kobatake [2], Yoshihiro Ito [1,3] and Hideyuki Miyatake [1,*]

[1] Nano Medical Engineering Laboratory, RIKEN Cluster of Pioneering Research, 2-1 Hirosawa, Wako, Saitama 351-0198, Japan.

[2] Department of Life Science and Technology, School of Life Science and Technology, Tokyo Institute of Technology, 4259 Nagatsuta-cho, Midori-ku, Yokohama 226-8503, Japan.

[3] Emergent Bioengineering Materials Research Team, RIKEN Center for Emergent Matter Science, 2-1 Hirosawa, Wako, Saitama 351-0198, Japan.

* Correspondence: mkawamot@riken.jp; Tel.: +81-48-467-2752, Fax: +81-48-467-9300 (M.K.) or miyatake@riken.jp; Tel.: +81-48-467-4979, Fax: +81-48-467-9300 (H.M.)

Analytical data
^{1}H NMR spectra

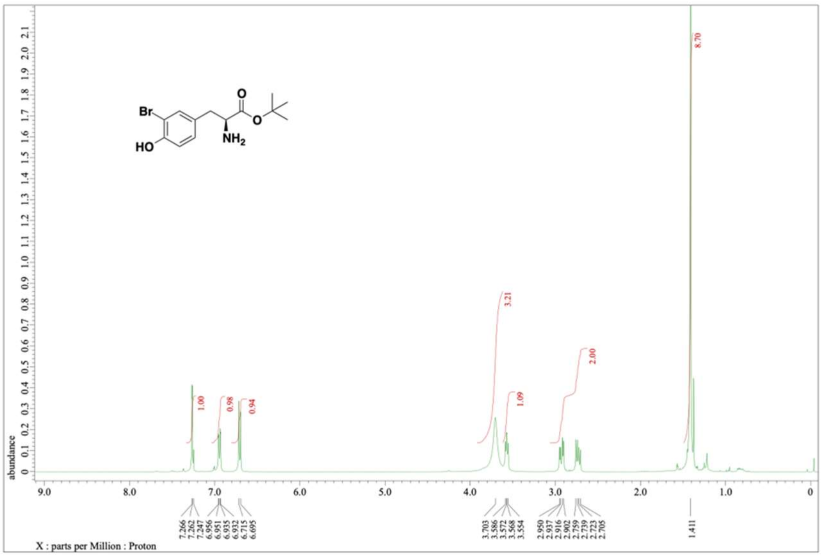

Figure S1. ^{1}H NMR spectrum of **3** in $CDCl_3$.

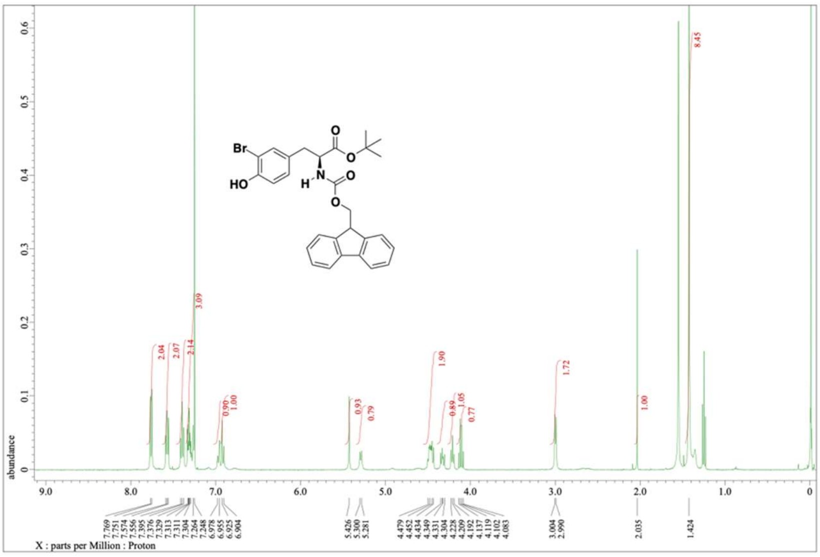

Figure S2. ^{1}H NMR spectrum of **4** in $CDCl_3$.

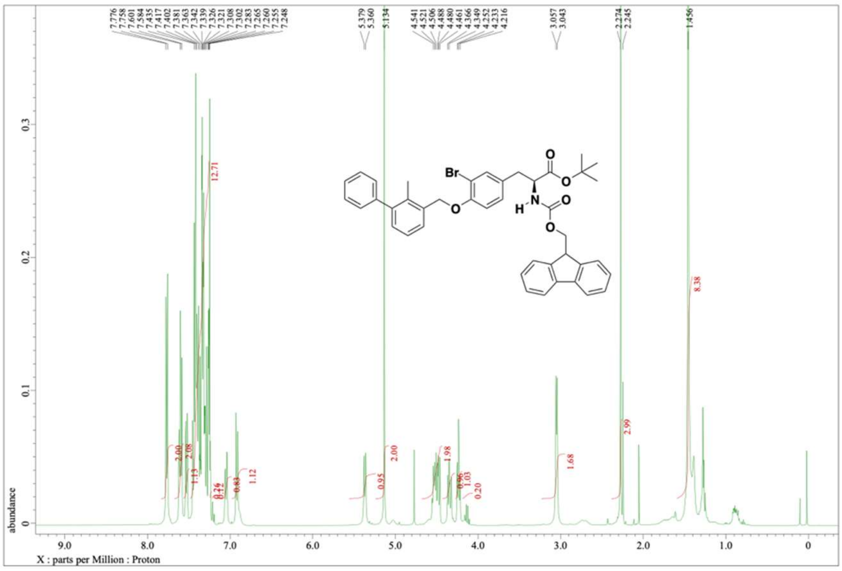

Figure S3. ^{1}H NMR spectrum of **5** in $CDCl_3$.

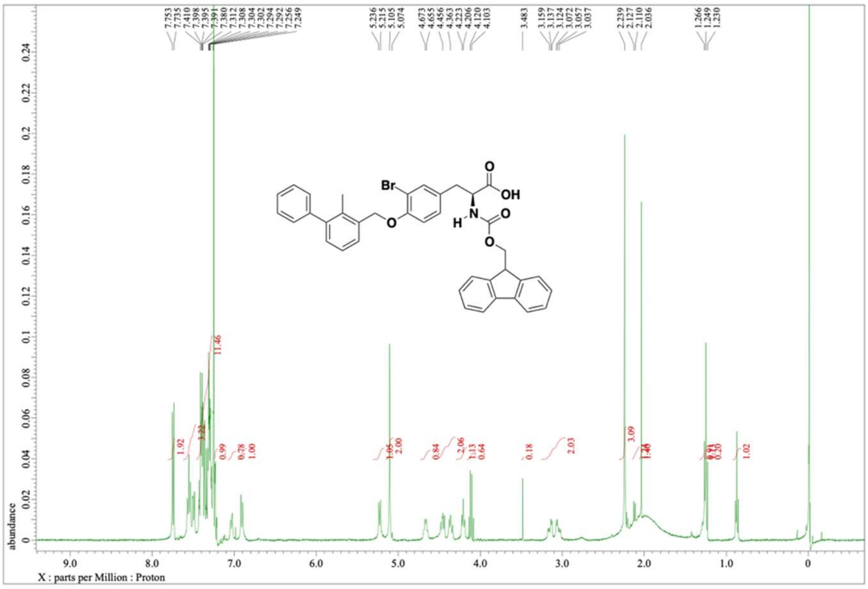

Figure S4. ^{1}H NMR spectrum of **6** in $CDCl_3$.

Table S1. Summary of molecular weights and HPLC elution times for the synthesized compounds.

Sequence	[a][M +H]$^+$	[b]t (min)	Sequence	[a][M +H]$^+$	[b]t (min)
X	440.1	15.8	GXG	554.1	13.0
GX	497.0	14.6	XNL	667.2	16.4
XG	497.1	18.8	XNH	691.2	14.9
XS	527.1	16.0	XHP	674.2	14.1
XR	586.1	18.2	XGG	554.1	16.4
XA	511.1	19.0	XCSE	759.4	15.1
XW	626.2	15.5	XGGG	611.1	19.3
YXC	706.2	17.3	WRXNN	1010.3	18.1
WXG	683.2	19.1	ERXNK	967.3	12.0
QXQ	696.2	14.4	WRXNQ	1024.4	13.4
NXR	710.2	18.2	XRRRR	1064.5	15.7
CXA	614.1	14.9	XGGGG	668.2	19.3
RXN	710.2	11.7	XGGGGG	725.3	18.4
SXR	683.2	18.4	CERXNKM	1201.4	15.6
CXR	699.2	14.8	FWRXNNI	1270.5	19.3

[a]Determined by MALDI-TOF MS. Matrix: 2,5-dihydroxybenzoic acid.

[b]Estimated by HPLC using an InertSustain C18 column and 35–65% acetonitrile containing 1% trifluoroacetic acid as the eluant. Letters represent the single letter amino acid code.

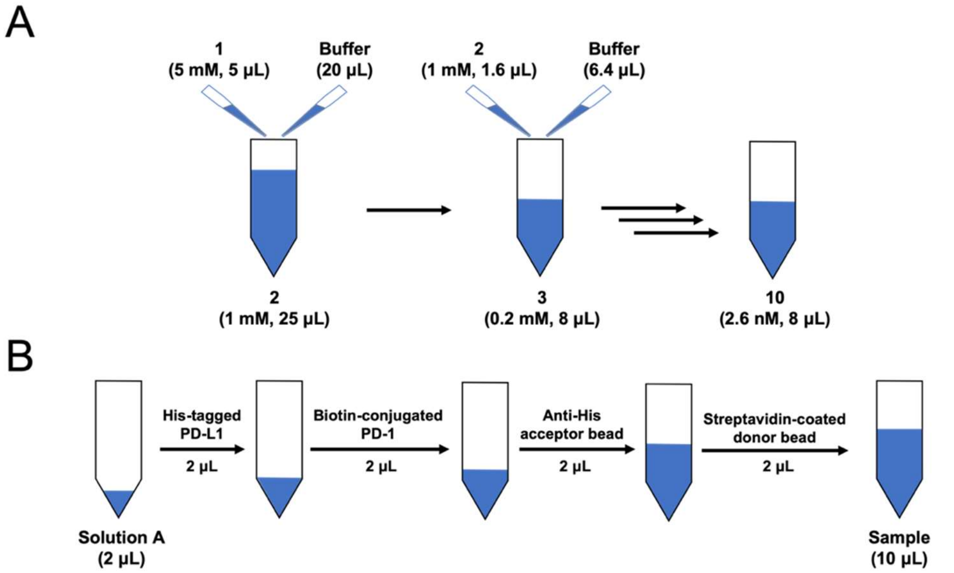

Figure S5. Preparation of (**A**) sample dilutions and (**B**) reaction samples.

Table S2. Concentrations of inhibitors in stock solutions (A) and in the assay sample.

	1	2	3	4	5	6	7	8	9	10
Solution A	5.0 mM	1.0 mM	0.2 mM	40 µM	8.0 µM	1.6 µM	0.32 µM	64 nM	13 nM	2.6 nM
Sample	1.0 mM	0.2 mM	40 µM	8.0 µM	1.6 µM	0.32 µM	64 nM	13 nM	2.6 nM	0.5 nM

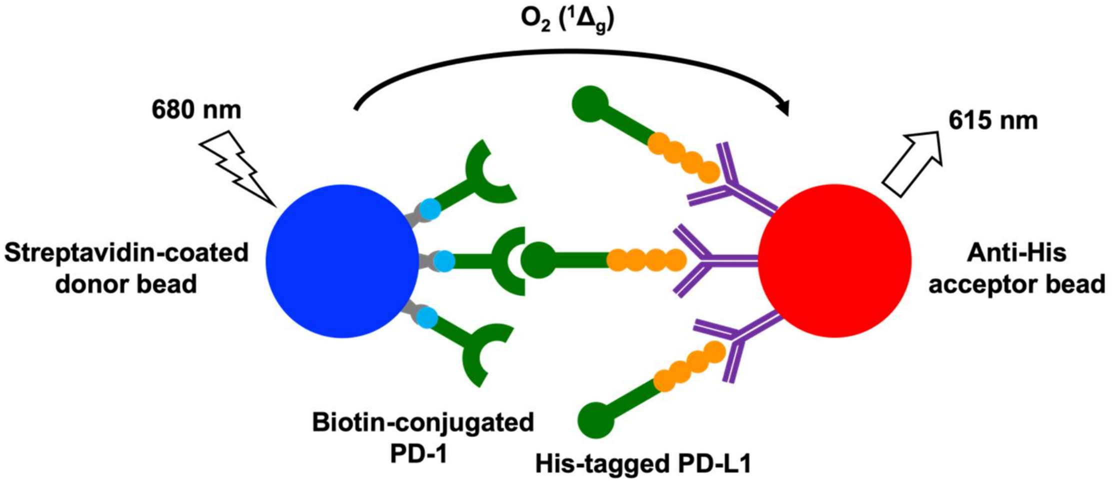

Figure S6. Principle of the Amplified Luminescence Proximity Homogeneous Assay (Alpha) assay for detection of PD-L1/PD-1 interactions through photoinduced energy transfer. Fluorescence intensity at 615 nm increases upon binding of PD-L1 to PD-1 and is reduced by competitive inhibition in the presence of **X** or **amino-X** compounds.
